# Resistance in *Pseudomonas aeruginosa*: A Narrative Review of Antibiogram Interpretation and Emerging Treatments

**DOI:** 10.3390/antibiotics12111621

**Published:** 2023-11-12

**Authors:** Federico Giovagnorio, Andrea De Vito, Giordano Madeddu, Saverio Giuseppe Parisi, Nicholas Geremia

**Affiliations:** 1 Department of Molecular Medicine, University of Padua, 35121 Padua, Italy; federico.giovagnorio@studenti.unipd.it (F.G.); saverio.parisi@unipd.it (S.G.P.); 2 Unit of Infectious Diseases, Department of Medicine, Surgery and Pharmacy, University of Sassari, 07100 Sassari, Italy; giordano@uniss.it; 3 Unit of Infectious Diseases, Department of Clinical Medicine, Ospedale “dell’Angelo”, 30174 Venice, Italy; 4 Unit of Infectious Diseases, Department of Clinical Medicine, Ospedale Civile “S.S. Giovanni e Paolo”, 30122 Venice, Italy

**Keywords:** *Pseudomonas aeruginosa*, multidrug resistance, β-lactams, antipseudomonal antibiotics, antibiotic resistance, emerging antibiotics

## Abstract

*Pseudomonas aeruginosa* is a ubiquitous Gram-negative bacterium renowned for its resilience and adaptability across diverse environments, including clinical settings, where it emerges as a formidable pathogen. Notorious for causing nosocomial infections, *P. aeruginosa* presents a significant challenge due to its intrinsic and acquired resistance mechanisms. This comprehensive review aims to delve into the intricate resistance mechanisms employed by *P. aeruginosa* and to discern how these mechanisms can be inferred by analyzing sensitivity patterns displayed in antibiograms, emphasizing the complexities encountered in clinical management. Traditional monotherapies are increasingly overshadowed by the emergence of multidrug-resistant strains, necessitating a paradigm shift towards innovative combination therapies and the exploration of novel antibiotics. The review accentuates the critical role of accurate antibiogram interpretation in guiding judicious antibiotic use, optimizing therapeutic outcomes, and mitigating the propagation of antibiotic resistance. Misinterpretations, it cautions, can inadvertently foster resistance, jeopardizing patient health and amplifying global antibiotic resistance challenges. This paper advocates for enhanced clinician proficiency in interpreting antibiograms, facilitating informed and strategic antibiotic deployment, thereby improving patient prognosis and contributing to global antibiotic stewardship efforts.

## 1. Introduction

*Pseudomonas aeruginosa*, a ubiquitous Gram-negative bacterium from the Pseudomonadaceae family, has garnered considerable attention for its resilience and adaptability in many environments. Notably, it bears a relatively larger genome, ranging from 5.5 to 7 Mbp, giving it a remarkable metabolic versatility and enabling it to thrive and adapt to diverse environmental shifts, thereby contributing to its survival in varied habitats, including water, soil, and associations with animals [1,2]. Furthermore, the bacteria’s widespread presence in water sources, such as tap water and hand soap dispensers, has been associated with hospital outbreaks, indicating a pressing need for stringent hygiene measures and environmental control [3,4]. *P. aeruginosa* genotypes analysis has identified household environments, such as sinks and nebulizers, as potential sources of infection, necessitating vigilant monitoring and sanitation [3,4].

While *P. aeruginosa’s* environmental tenacity is noteworthy, its role as an opportunistic pathogen has raised significant concerns, particularly in hospital settings [5]. It is notorious for causing nosocomial infections and ventilator-associated pneumonia, primarily affecting immunocompromised individuals, severe burn victims, and patients with underlying health conditions such as cystic fibrosis (CF) and chronic obstructive pulmonary disease (COPD) [6,7,8,9,10]. In particular, *P. aeruginosa*’s ability to form biofilms, coupled with its intrinsic, acquired, and adaptive resistance mechanisms, has rendered it a formidable adversary in the clinical setting [11,12,13,14]. It exhibits resistance to many antibiotics, including aminoglycosides, fluoroquinolones, and β-lactams, through mechanisms such as low outer membrane permeability, the expression of efflux pumps, and the production of antibiotic-inactivating enzymes [11,15,16,17]. Acquired resistance through horizontal gene transfer and mutations, along with adaptive resistance exemplified by biofilm formation and the emergence of persister cells, further complicate its treatment landscape [18].

Globally, the incidence of multidrug-resistant (MDR.) *P. aeruginosa* has exhibited an alarming upswing, posing considerable challenges to public health and clinical treatment [19,20]. The European Centre for Disease Prevention and Control (ECDC) has reported a varying prevalence of MDR *P. aeruginosa*, with some regions witnessing a heightened occurrence, underscoring the geographical disparity and the necessity for region-specific interventions [21]. The global landscape is similarly marked by a heterogeneous distribution of MDR strains, emphasizing the criticality of continuous surveillance and adaptive strategies to curb the spread of resistance [22]. The escalating challenge posed by *P. aeruginosa* has been accentuated by the World Health Organization (WHO), which has categorized carbapenem-resistant strains as being in critical need of new antibiotics [23]. The emergence of these strains has been associated with elevated morbidity and mortality, amplifying the urgency for innovative and effective therapeutic approaches [24]. These strategies include novel antibiotics and non-antibiotic therapeutic options, including phage therapy, nanoparticle application, and quorum sensing inhibition [25,26,27,28,29,30,31]. These strategies aim to augment or substitute conventional antibiotic treatments in addressing the rising tide of antibiotic resistance.

In prescribing antibiotics, it has become fundamental to discern the potential resistance mechanisms inherent in *P. aeruginosa*, enabling clinicians to determine the most effective antibiotic treatment for this type of infection. A pivotal tool in this endeavor is the analysis of antibiograms, as they reveal the sensitivity patterns of the bacteria, providing invaluable insights into the underlying resistance mechanisms. By meticulously examining these patterns, clinicians can infer the probable resistance strategies employed by *P. aeruginosa*, thereby guiding a more informed and targeted approach to antibiotic therapy, optimizing antibiotic stewardship, and improving patient outcomes.

## 2. Materials and Methods

A comprehensive literature search was conducted to identify relevant studies concerning the principal mechanisms of resistance in *P. aeruginosa*. The search strategy was implemented using online databases (Pubmed/MEDLINE), books written by experts in the fields of microbiology, infectious diseases, and ICU, and antibiograms collected from real-life cases. The search was not restricted by language or publication date and covered articles up to the cutoff date of October 2023. The following keywords and MeSH terms were used: “*Pseudomonas aeruginosa* AND mechanisms of resistance”, “Therapy strategies AND Pseudomonas aeruginosa”, “Antibiotic resistance AND Pseudomonas aeruginosa”, “Efflux pumps”, “Systematic review AND Pseudomonas aeruginosa”. Studies were included in this narrative review if they met the following criteria: studies reporting the in vitro activity of antibiotics active against *P. aeruginosa*; reviews reporting the mechanism of resistance of *P. aeruginosa*; studies reporting the prevalence of susceptibility and resistance to certain classes of antibiotics.

The search strategy had no time limits or language restrictions. We screened the articles by title, abstract, and full text. After an initial screening of titles and abstracts of published articles, the reviewers evaluated full articles to assess eligibility for each study’s inclusion in this narrative review. A study was included if it was likely to provide valid and valuable information according to the review’s objective.

## 3. Mechanisms of Resistance

### 3.1. Porins

The mechanisms of resistance in *P. aeruginosa* are various, with one of the most crucial being related to the permeability of the outer membrane and the efflux systems [32]. The *P. aeruginosa* outer membrane is highly restrictive, and this is due to its lower folder [33,34] and the regulated expression of specific outer membrane porins. One of those porins, OprF, exists in two conformations: the most frequent form, where the dominant structures of the porin are closed, and one where OprF exhibits open channels, which account for about 5% of this protein [35]. This system is unbalanced in favor of reduced permeability. Additionally, the loss of OprF is associated with biofilm formation [36], which gives bacteria a reduced susceptibility to antimicrobial agents, even without mechanisms such as enzymes or efflux pumps [37].

Another significant porin in *P. aeruginosa* is OprD, which plays a role in antibiotic uptake due to the carbapenem binding site, thereby increasing antibiotic resistance (Table 1) [38]. Specifically, gene mutations lead to a down-regulation in the expression of OprD, which typically gives resistance to imipenem (IMP) [39]. The susceptibility of meropenem (MEM) is variable, depending on the type of OprD gene mutations [40]. Furthermore, strains of *P. aeruginosa* with mutations in the OprD gene may exhibit elevated cefiderocol (FDC) minimum inhibitor concentration (MIC) [41].

*P. aeruginosa* can also express other porins, like OprH, the smallest produced. Its overexpression is linked to Mg^2+^ starvation, which confers modification and stabilization of the lipopolysaccharide (LPS) and ultimately confers resistance to Polymyxin B and Gentamicin (GM) [42,43].

**Table 1:** Phenotypic antibiogram of clinical strain *P. aeruginosa*, showing an OprD loss of function/downregulation.

*P. aeruginosa*	Blood Culture	
Antibiotic	MIC	SIR *
Meropenem/Vaborbactam	=4/4	S
Imipenem/Relebactam	=2/2	S
Amikacin	=8.0	S
Cefepime	=4.0	I
Ceftazidime	=4.0	I
Ceftazidime/Avibactam	=2.0	S
Ceftolozane/Tazobactam	=1.0	S
Ciprofloxacin	=0.125	I
Colistin	=1.0	S
Imipenem	>8	R
Meropenem	=4	I
Piperacillin/Tazobactam	=8.0	I

MIC = minimum inhibitory concentration; SIR = Susceptible (S), Susceptible, Iincreased Eexposure (I), and Resistant (R). * Breakpoint sec. Clinical breakpoints (v 13.1) [44]. Comment: phenotype resistance to imipenem, increased MIC of meropenem, and main-tained susceptibility to others common β-lactams. The phenotype could be compatible with OprD loss of functions/down-regulation.

### 3.2. Efflux Pump Systems

The efflux systems of *P. aeruginosa* play a pivotal role, representing a significant resistance mechanism. Six families of efflux pumps have been described in the literature: the small multidrug resistance (SMR) family; the major facilitator superfamily (MFS); the resistance/nodulation/cell division (RND) family; the ATP-binding cassette (ABC) superfamily; the multidrug and toxic compound extrusion (MATE) family [45,46]; and the proteobacterial antimicrobial compound efflux (PACE) family [47]. The co-expression of multiple pumps can lead to the MDR phenotype. In particular, the proteins belonging to the RND family are the most important for antibiotic resistance in *P. aeruginosa* [48]. These are called multidrug efflux (Mex) pumps, including MexXY, MexAB-OprM, MexCD-OprJ, and MexEF-OprN, all genetically encoded [49].

The Mex are composed of three distinct proteins: a periplasmatic adaptor protein, such as MexA, MexX, MexC, or MexE; a resistant-nodulation-cell division transporter (RNDt), like MexB, MexY, MexD, or MexF; and a channel-forming outer membrane factor (OMF), such as OprM, OprJ, or OprN (Table 2) [50]. Wild-type (WT) *P. aeruginosa* strains constitutively express MexAB-OprM, and its overexpression is correlated with resistance to various antibiotic classes, resulting in numerous phenotypes, including carbapenem-resistant *P.* *aeruginosa* (CRP) [51].

MexAB-OprM displays the broadest substrate profile among the efflux pump systems and, when overexpressed, can export fluoroquinolones (FQ) and almost all β-lactams (BL) (except IMP), including MEM (Table 3) [50,52,53,54].

Other antibiotics affected by MexAB-OprM are macrolides, tetracyclines, lincomycin, and chloramphenicol (14). Moreover, it has been shown that the overexpression of MexAB-OprM and AmpC, a chromosomally encoded C β-lactamase, has synergistic effects on the resistance of *P. aeruginosa* to most antipseudomonal BL, except for ceftolozane/tazobactam (TOL-TZB), IMP, and imipenem/elebactam (IMP-REL) [55]. It is also known that the overexpression of MexAB-OprM could contribute to FDC resistance [41]. The overexpression of MexAB-OprM and the downregulation of OprD are among of the most relevant causes of clinical resistance to MEM [54].

The MexXY efflux pump is the only one in *P. aeruginosa* that does not contain a coding sequence for an outer membrane factor, but usually forms a multidrug efflux pump with the OprM of the MexAB complex [56]. Its expression is inducible by antimicrobials, and it is regulated mainly by two regulatory systems [51]. Its role in antibiotic resistance is well known, especially for aminoglycosides [57]. MexXY overexpression is commonly paired with the expression of aminoglycoside-modifying enzymes (AMEs). These two mechanisms synergize, ultimately providing resistance to aminoglycosides (AMG) [58]. MexXY is also associated with resistance to most antipseudomonal antibiotics [53]. Furthermore, mutations occurring in the MexY are linked to resistance to FQ, AMG, and cefepime (FEP) [59].

The overexpression of MexAB-OprM and MexXY is frequent among clinical strains, accounting for up to 30% of cases (Table 4) [54]. MexCD-OprJ is a multidrug efflux pump encoded by an operon, typically silent or expressed at a low level in *P. aeruginosa* [60]. Several mutations can trigger the overexpression of this pump, generating multiple antibiotic resistance actions [61]. In this scenario, the main antibiotics affected are Penems and FQ, such as Ciprofloxacin and Levofloxacin [59,61]. Other antipseudomonal BL, like FEP, may also be impacted [62].

It is essential to highlight that mutations that occur in MexD can change the substrate of the efflux pump, resulting in resistance to carbenicillin [63] and ceftazidime-avibactam (CAZ-AVI) [64]. TOL-TZB is an important option against MDR *P. aeruginosa* and is typically unaffected by the efflux system’s substrate [64]. However, in the case of MexD mutation, TOL-TZB susceptibility may also be compromised [64].

MexEF-OprN, like MexCD-OprJ, is usually inactive, but in some circumstances, gene mutations can overexpress this efflux pump system. Its overexpression is associated with resistance to chloramphenicol, FQ, and trimethoprim [65]. However, the MexEF-OprN alone is not particularly relevant to confer an MDR profile to *P. aeruginosa*. Its overexpression is linked to genes that are involved in the downregulation of OprD [65], granting resistance to carbapenems [66,67] and Colistin (COL) [68].

**Table 2:** Efflux pump systems and their activity versus antibiotics.

Efflux System	Antibiotics Affected by Altered Expression
MexAB-OprM	Aztreonam, β-lactams ^a^, macrolides, tetracyclines, lincomycin, chloramphenicol, fluoroquinolones [11,50,54], higher in MIC or resistance to relebactam [69] could raise MIC of cefiderocol ^b^ [41].
MexXY-OprM	Aminoglycosides, cefepime, fluoroquinolones, tetracyclines and macrolides [53,59,69].
MexCD-OprJ	Fluoroquinolones, penems, and cefepime [59,61,62], carbanecillin, piperacillin, ceftolozane/tazobactam, and ceftazidime/avibactam ^c^ [63,64].
MexEF-OprN	Chloramphenicol, fluoroquinolones, trimethoprim, colistin, and imipenem ^d^ [65,66,67,68].

^a^ MexAB-OprM exports all β-lactams, except imipenem. ^b^ The overexpression of MexAB-OprM alone cannot provide straightforward resistance to cefiderocol. ^c^ Resistance to carbanecillin, piperacillin, ceftolozane/tazobactam, and ceftazidime/avibactam occurs with a specific mutation for MexD. ^d^ Resistance to imipenem due to the contemporary downregulation of OprD.

**Table 3:** Phenotypic antibiogram of clinical strain *P. aeruginosa*, compatible with an overexpression of MexAB-OprM.

*P. aeruginosa*	Blood Culture	
Antibiotic	MIC	SIR *
Aztreonam	>16	R
Amikacin	≤8.0	S
Tobramycin	≤2	S
Cefepime	=8	I
Ceftazidime	=8	I
Ceftazidime/Avibactam	=4.0	S
Ceftolozane/Tazobactam	=1.0	S
Ciprofloxacin	=0.125	I
Colistin	=1.0	S
Imipenem	≤1	I
Meropenem	=4	I
Piperacillin/Tazobactam	=64	R

MIC = minimum inhibitory concentration; SIR = Susceptible (S), Susceptible, Iincreased Eexposure (I), and Resistant (R).* Breakpoint sec. Clinical breakpoints (v 13.1) [44]. Comment: phenotype resistance to aztreonam, ticarcillin and piperacillin/tazobactam, susceptibility of aminoglycosides, increased MIC to cephalosporines of third and fourth generations and to meropenem. This phenotype is compatible withto an overexpression of MexAB-OprM.

**Table 4:** Phenotypic antibiogram of clinical strain *P. aeruginosa*, compatible with an overexpression of MexXY-OprM, probably associated with the overexpression of OprH.

*P. aeruginosa*	Blood Culture	
Antibiotic	MIC	SIR *
Aztreonam	≤8	S
Amikacin	>16	R
Tobramycin	>2	R
Cefepime	16	R
Ceftazidime	≤2	I
Ceftazidime/Avibactam	≤2.0	S
Ceftolozane/Tazobactam	≤1.0	S
Ciprofloxacin	>0.5	R
Levofloxacin	>2	R
Colistin	>4	R
Imipenem	≤1	I
Meropenem	≤0.125	S
Piperacillin/Tazobactam	8	I

MIC = minimum inhibitory concentration; SIR = Susceptible (S), Susceptible, Iincreased Eexposure (I), and Resistant (R).* Breakpoint sec. Clinical breakpoints (v 13.1) [44].Comment: phenotype resistance to aminoglycosides, cefepime, fluoroquinolones and colistin, susceptibility to other common β-lactams. This phenotype is compatible with an overexpression of MexXY-OprM associated with the overexpression of OprH, which is notable due to the resistance to colistin.

### 3.3. Penicillin-Binding Protein Mutations

Penicillin-binding proteins (PBPs) are a group of periplasmatic enzymes involved in the polymerization, cross-linking, and modification of bacterial peptidoglycan [70,71]. PBPs are classified by their molecular weight into High-Molecular-Mass (HMM) and Low-Molecular-Mass (LMM) [72,73]. The HMM PBPs are defined as “essential penicillin-binding proteins”, playing a crucial role in the lifecycle of the bacteria by acting in the final phase of the peptidoglycan synthesis. These included PBP1_a_/PBP1_b_, PBP2, and PBP3 [73,74]. They are further classified into Class A (e.g., PBP1) and Class B (e.g., PBP2 and PBP3) [72].

The LMM PBPS are PBP4, PBP5, and PBP7 [72,73], further divided into Class C PBPs [72]. Research focusing on the correlation between PBPs and antibiotic resistance, particularly in *P. aeruginosa*, has not fully elucidated the role of LMM PBPs in antibiotic activity. For instance, PBP5 has demonstrated direct β-lactamase activity in *P. aeruginosa* [75]. Mutations in the nonessential *dacB* gene, which encodes PBP4, are associated with AmpC-overexpression, leading to high-level β-lactams (BL) resistance [73,76], specifically reducing the susceptibility of piperacillin (PIP), cefotaxime (CTX), CAZ, FEP, and aztreonam (AZT) [76]. Moreover, not all mutations occurring in genes that encode PBP4 have the same impact on the overexpression of AmpC. In detail, the DacB-DacC-PbpG triple mutant has been linked to the highest basal and induced *ampC* expression levels, ultimately leading to more resistance to BL [77]. Mutations occurring in PBP3 result in resistance to CAZ, FEP, PIP-TZB, TOL-TZB, CAZ-AVI, and MEM [73].

Finally, LMM PBPs play a role in the intrinsic resistance to BL regardless of the relationship with AmpC overexpression [77].

Another important aspect is the ability of the various BL antibiotics to bind to the PBPs, both in number and tightness. The carbapenem IMP exhibits the most significant activity against *P. aeruginosa* due to the number and affinity of the PBPs it binds to. In contrast, TOL shows a marked binding affinity to PBP3 [73]. Furthermore, a correlation has been observed between the relative binding affinity of specific PBPs for a particular antipseudomonal agent. This correlation is influenced by the relative abundance of each PBP, depending on the expression and the absence of others [74]. For instance, the absence of PBP1_a_ leads to a higher affinity of CAZ, TOL, and IMP to PBP1_b_. Conversely, some strains of Pan-BL-resistant *P. aeruginosa* exhibit a 50% increase in inhibitory concentrations (IC_50_s) when compared to susceptible strains for IMP-binding PBP2 and CAZ-, TOL-, and IMP-binding PBP3 [74]. Despite ongoing research, a clear correlation between certain PBP modifications (such as PBP5 or PBP7) and the emergence of a frank BL resistance has not been established. This suggests that PBPs are part of a more complex system influencing antibiotic resistance [74,78].

### 3.4. Antibiotic Inactivating Enzymes

#### 3.4.1. β-Lactamases

β-lactamases are enzymes that inactivate BL antibiotics by cleaving the amide bond of the BL ring. They are categorized into four classes according to Ambler’s classification, based on their amino-acid sequence similarity [79]. Classes A, C, and D utilize serine for substrate hydrolysis, while class B enzyme hydrolysis BL involves a metal ion, most commonly a zinc ion [80,81]. Classes of β-lactamases are summarized in Table 5. β-Lactamases are encoded by either chromosomal genes or transferable genes located on plasmids and transposons. Moreover, β-lactamase genes (bla) frequently reside on integrons, which often carry multiple resistance determinants. When mobilized by transposable elements, integrons can facilitate the further dissemination of MDR among different bacterial species [82].

β-lactam inhibitors (BLI) diminish the effectiveness of β-lactamases. This class includes clavulanic acid (CLA), sulbactam (SUL), TZB, AVI, Vaborbactam (VAB), and Relebactam (REL) [83,84]. AVI is active against classes A, C, and some enzymes of class D β-lactamases; VAB can inhibit class A β-lactamases and REL for Class A and Class C enzymes [84].

Only one BL has a high stability against hydrolysis by most beta-lactamases, including classes A, B, C, and D. Therefore, FDC represents a valid option against the *P. aeruginosa* XDR stain [85].

#### 3.4.2. Class A β-Lactamases

*P. aeruginosa* can acquire additional β-lactamase through horizontal gene transfer, particularly class A enzymes such as sulfhydryl variable (SHV), Temoniera (TEM), cefotaxime-M (CTX-M), Belgium extended β-lactamase (BEL), Pseudomonas extended resistant (PER), Vietnam extended-spectrum β -lactamase (VEB), Klebsiella pneumoniae carbapenemase (KPC), and Guiana extended-spectrum β-lactamases (GES) families [86,87,88,89,90,91]. A novel class A carbapenemase, German Pseudomonas carbapenemase (GPC-1), was recently discovered in *P. aeruginosa* [92].

The most important Extended-Spectrum β-Lactamases (ESBLs) in *P. aeruginosa* include TEM, SHV, CTX-M, BEL, PER, and VEB (Table 6) [93]. ESBLs can hydrolyze extended-spectrum cephalosporins and AZT, but they do not degrade second-generation cephalosporins (for example, cefoxitin). CLA, SUL, TZB, AVI, VAB, and REL can inhibit these enzymes [90]. Among the PER family, PER-1 and PER-2 are the most common and are less inhibited by CLA, TZB, and AVI compared to other class A β-lactamases [94]. ESBL-producing strains typically conserve sensitivity to IMP, carboxypenicillins plus BLI, and ureidopenicillin plus BLI.

The identification of ESBL can be conducted with phenotypic screening tests using the Kirby–Bauer method with cephalosporin (for example, with CAZ) and monobactam discs [93,95]. Confirmation tests rely on the synergy between cephalosporins and CLA. One of the methods used is the combined disc method, which compares the diameters of the inhibition areas of a cephalosporin disc and a cephalosporin plus CLA disc. If the stain produces ESBL, the inhibition area of the disc containing CLA is at least 5 mm larger than the inhibition area of the disc without [93]. The synergy test can also be performed with an E-test (having CAZ/CTX on one half and CAZ/CTX plus CLA on the other half) or with ESBL-AGAR medium.

Among the GES enzymes, only variants with alterations at amino acid 170, changing glycine to asparagine or serine with a polar uncharged side chain, can degrade cephalosporins like CAZ, FEP, AZT, carbapenems [96], and TOL-TZB [97].

Isolates of *P. aeruginosa* strains with different ESBL expressions are summarized in Table 6. The antibiogram compatible with GES expression is reported in Table 7.

KPC is a potent serine carbapenemase encoded by the bla_KPC_ gene. In 2007, an isolate of *P. aeruginosa* exhibiting high resistance to carbapenems and harboring bla_KPC_ was reported in Medellin, Colombia [98]. Since then, additional reports of such isolates have been reported, mainly in the Americas and Asian countries [99]. KPC confers a broad antibiotic resistance spectrum, having the capability to hydrolyze broad-spectrum penicillins, oxymino-cephalosporins, cephamycins, and carbapenems [97]. New BLI, such as AVI, REL, and VAB, can inhibit KPC [100] enzymes.

**Figure 1:**
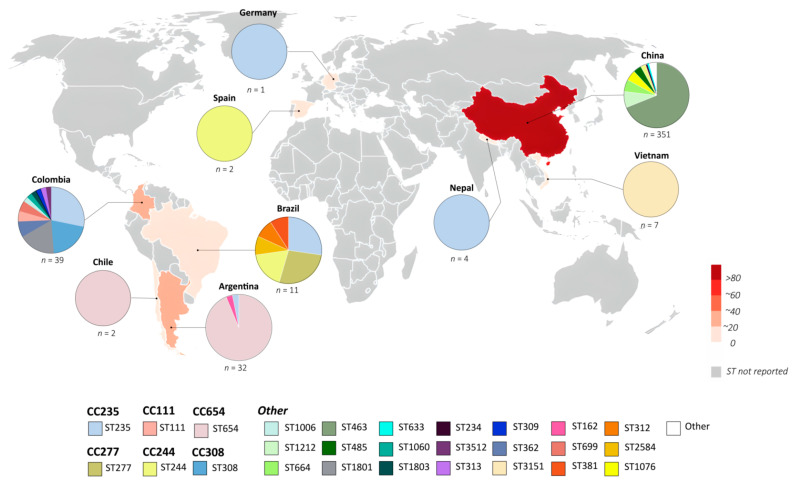
Worldwide distribution of the *P. aeruginosa* blaKPC isolates [105,106].

The global distribution of KPC is illustrated in Figure 1.

Phenotypic detection could be aided by screening tests that evaluate the growth of *P. aeruginosa* in the presence of MEM or IMP. Confirmation phenotypical tests use MEM discs and MEM plus boronic acid discs. A 5 mm or larger diameter between the MEM disc and the MEM plus boronic acid disc proves that the strain produces a class A carbapenemase [84,101]. Genotypic approaches with polymerase chain reaction (PCR) can facilitate direct carbapenemase-type detection with low costs and resource utilization [102,103].

Another class A β-lactamase is PIB-1, encoded in the core genome, and it can degrade IMP [104].

#### 3.4.3. Class B β-Lactamases

Metallo- β-lactamases (MBL) are subclassified into B1, B2, and B3, based on functional and structural factors [107,108]. The dominant subclass in *P. aeruginosa* is B1, which contains the most significant number of relevant acquired MBL enzymes. Class B β-lactamases utilize Zn^2+^ atoms for hydrolysis [108,109]. MBL activity is reduced in the presence of divalent ion chelators, such as ethylenediaminetetraacetic acid (EDTA) and dipicolinic acid [93,107]. The MBL detection is based on a 5 mm or larger difference between the MEM disc and the MEM plus EDTA disc [110]. Disc tests and the E-test^®^ have been used for several decades, but have poor specificity and no robust data supporting their routine clinical use. Colorimetric tests probably offer the best-proven specificity at the moment. Still, no test appears sufficiently specific for routine use in clinical practice. Therefore, PCR should be preferred to detect carbapenemases (Table 8) [102,103].

MBLs hydrolyzed a broad spectrum of BL, such as penicillins, cephalosporins, and carbapenems, but do not affect monobactams. Additionally, BLIs do inhibit this class of enzymes [111]. FDC could be a valid option in MBL-producing *P. aeruginosa* strains [112,113]

Class B enzymes are the most prevalent carbapenemases produced by *P. aeruginosa*. Verona Integron-encoded MBL (VIM) and Imipenemases (IMP) are, respectively, the first and the second most common MBLs found in clinical isolates. New Delhi MBL (NDM) has also been identified in various *P. aeruginosa* isolates. Numerous other enzymes such as Australian imipenemase (AIM), Central Alberta M.B.L. (CAM), Dutch imipenemase (DIM), Florence imipenemase (FIM), German imipenemase (GIM), Hamburg M.B.L. (HMB), São Paulo M.B.L. (SPM), and Seoul imipenemase (SIM) have been reported [107].

#### 3.4.4. Class C β-Lactamases

*P. aeruginosa* possesses a chromosomally encoded class C β-lactamase known as AmpC [114]. These enzymes are resistant to penicillins (PIP) and cephalosporins (CAZ, cefoxitin). They are not susceptible to BLI, such as CLA [115]. AmpC has a limited hydrolysis activity against FEP and is inhibited by cloxacillin and oxacillin [116]. AmpC does not affect carbapenems [117]. The cefoxitin sensitivity reduction represents the phenotypic non-standard screening method. Other phenotypic methods for identifying AmpC involve the use of cloxacillin and boronic acid [93]. Table 9 shows a possible antibiogram with AmpC expression.

AmpC enzymes’ regulation is mediated by the transcriptional regulator AmpR. During peptidoglycan synthesis, the uridine diphosphate–N-Acetylmuramic acid (UDP-NAM) pentapeptide binds to AmpR, resulting in a complex AmpR-AmpC that inhibits the transcription of AmpC. Peptidoglycan fragments are processed for recycling by NagZ, AmpD, and other enzymes.

The inhibition of PBPs increases the intracellular concentrations of the N-Acetylglucosamine (NAG)-NAM pentapeptide, NAM pentapeptide, and NAM tripeptide that saturate AmpD, raising the intracellular concentration of the AmpR-activating compounds causing the expression of AmpC. PBPs, particularly the PBP4 protein, can influence the cytoplasmic concentration of NAG-NAM. The depression of PBP4 levels is a significant inducer of AmpC expression [77]. Mutations in dacB, encoding PBP4, are common in BL-resistant isolates and can contribute to AmpC overexpression [74]. PBP4 inhibition also activates the CreBC signaling, a global regulator of bacterial fitness, biofilm development, and AmpC expression [118]. AmpR mutants contribute to BL resistance, inhibiting the UDP-NAM from binding to AmpR, leading to the constitutive expression of AmpC at high levels [119]. The *P. aeruginosa* strain with increasing BL resistance was also found in AmpD mutations [80].

AmpC variants can be hundreds, with unusual substrate specificities. They are considered the leading cause of resistance of clinical strains of *P. aeruginosa* to antipseudomonal penicillins and cephalosporins. These mutants are known as extended-spectrum AmpC (ESAC) or Pseudomonas-derived cephalosporinase (PDC). Additionally, AmpC mutations can reduce the affinity of inhibitors such as AVI, TZB, and REL for AmpC [80,120,121]. Some AmpC mutants in Enterobacterales can also increase in FDC MICs by >32-fold, and this variant may contribute in part to the FDC-resistant *P. aeruginosa* phenotypes. The presence of some PDC with substitutions in the region of the AmpC omega loop contributes to reducing the activity of TOL-TZB, CAZ-AVI, and FDC. However, these strains potentially increase susceptibility to IMI-REL [122,123].

Transferable Class C enzymes, originating from chromosomal enzymes transferred to mobile elements, are relatively rare, but some have emerged through horizontal gene transfer, including FOX-4 and CMY-2 [80].

An example of an antibiogram with AmpC mutants is reported in Table 10.

#### 3.4.5. Class D β-Lactamases

Class D β-lactamases encompass the OXA family, so named due to their high activity against oxacillin [124]. This superfamily of serine β-lactamases can degrade all BL [125]. OXA-10-like was the first enzyme of this class discovered in *P. aeruginosa*. It presented the ability to confer resistance to CAZ [126]. In a recent study, OXA-like enzymes showed higher prevalence, with 15.4% of *P. aeruginosa* isolates harboring OXA β-lactamases [127]. In *P. aeruginosa*, isolates have also been detected of OXA-9, which have the uncommon property of being inhibited by CLA and cloxacillin [128], OXA-13, OXA-17, OXA-18, OXA-20 [129], and OXA-31, which possesses the ability to hydrolyze FEP and cefpirome slightly [130].

Carbapenem-hydrolyzing Class D (CHDL) enzymes have relatively low activity against carbapenems and are not effectively inhibited by EDTA or CLA. In *P. aeruginosa*, the principal CHDL identified belonged to groups such as OXA-40-like, OXA-48-like, OXA-50-like, and OXA-198-like [107,131]. Less common CHDLs, such as OXA-23-like, have been associated with MDR phenotypes.

Certain enzymes can impact the susceptibility of newer anti-MDR cephalosporins. Enzymes such as OXA-2, OXA-46 and four variants of the OXA-10 subgroup can cause an 8-fold to 32-fold increase in FDC MICs. In *P. aeruginosa* encoding OXA-19 (derived from the OXA-10 subgroup), there is a significant reduction in the activity of CAZ, CAZ-AVI, TOL-TZB, and FDC. New CHLDs, such as OXA-539, can determine resistance to third–fourth generation cephalosporins, CAZ-AVI and TOL-TZB [132]

### 3.5. Interference with Antibacterial Agents

Modifying the antibiotic target is one of the most common strategies bacteria employ to evade the effects of antimicrobials. FQ inhibits bacterial DNA replication by targeting DNA gyrase and topoisomerase IV. Mutations in gyrA, gyrB (for DNA gyrase), parC, and par E (for topoisomerase IV) cause a reduced affinity for FQ to these two proteins [11].

Resistance to FQ can also be plasmid-mediated (PMQR). Three PMQR-mediated mechanisms were recognized, including qnr genes, the acetyltransferase aac(6′)-Ib-cr, and active efflux pumps such as QepA and OqxAB10,11 [133]. The qnr genes interfere with the binding of FQ to their protein targets [134].

Ribosomal mutations contribute significantly to resistance against aminoglycosides and polymyxins. Polymyxin resistance in *P. aeruginosa* was associated with the modification of the polymyxin-binding partner LPS by the addition of 4-amino-L-arabinose (L-Ara4N) to the phosphate groups within the lipid A moiety of LPS. Moreover, mutations in PhoPQ and PmrAB regulatory systems promoted the modification of aminoarabinose addition to lipid A, leading to enhanced Polymyxin resistance [11].

### 3.6. Fosfomycin Resistance Mechanisms

Fosfomycin (FOS) is an old antibiotic with activity against both Gram-positive and Gram-negative bacteria, including *P. aeruginosa* [135]. FOS is transported into the bacterial cells via GlpT and UhpT, glycerol-3-phosphate and glucose-6-phosphate symporters, respectively. Once inside the cell, FOS inhibits MurA activity, a crucial enzyme involved in transferring phosphoenolpyruvate (PEP) to the 30-hydroxyl group of UDP-N-acetylglucosamine, an initial step for bacterial cell wall biosynthesis [136,137,138]. A peculiarity of this drug is its enhanced efficacy under anaerobic conditions, unlike many antibiotics, whose activity diminishes in such environments [139,140,141,142].

*P. aeruginosa* harbors the gen *fosA* in the chromosome, encoding an enzyme that inactivates FOS [143,144]. Notably, *P. aeruginosa* lacks the genes encoding for the UhpT transporter, making FOS uptake solely dependent on the GlpT transporter [145,146]. However, *P. aeruginosa* infections often occur in oxygen-reduced environments, primarily when biofilm production occurs [147,148]. In these conditions, FOS is more active against *P. aeruginosa* [142]. In treating *P. aeruginosa* infections, FOS can be used in combination therapy due to its synergistic activity with lots of antipseudomonal antibiotics [149,150,151]. Furthermore, it is an essential drug against MDR *P. aeruginosa* due to its unique structure, which is dissimilar to other antipseudomonal agents, allowing it to retain its activity [152].

## 4. New Treatment Options

Managing *P. aeruginosa* infections has always been a clinical challenge due to its intrinsic and acquired resistance mechanisms. Traditionally, the primary treatment approach was monotherapy with antipseudomonal agents such as FQ, PIP-TZB, or carbapenems [153,154,155]. However, the emergence of MDR strains has prompted a shift towards combination therapy [156]. Due to their synergistic effects, common combinations include β-lactam agents with aminoglycosides or FQ [157]. Another reason for using combination therapy is to counter the bacteria’s ability to form biofilms, which are inherently resistant to many antibiotics. It is also crucial to consider the phenomenon of inducible resistance, where the bacteria can upregulate specific resistance genes in response to certain antibiotics [158]. This review will further discuss the different types of resistance mechanisms.

The rise of MDR strains has necessitated the development of new antibiotics. Notable additions to the therapeutic arsenal include TOL-TZB, CAZ-AVI, IMI-REL, and FDC.

TOL-TZB (Zerbaxa^®^) is a novel antibiotic combination with a next-generation cephalosporin and TZB, a suicidal BLI [159]. This combination has an enhanced affinity for PBPs and potent activity against *P. aeruginosa*, including ESBL and AmpC-producing strains [160]. Clinical studies have emphasized its efficacy against Pseudomonas infections, especially in complicated urinary tract (cUTI) and intra-abdominal infections (cIAI) [161]. Caston et al. treated 20 infections caused by *P. aeruginosa* MDR with TOL-TZB, which included 12 cases of septic shock, 6 cases of pneumonia, 1 case of otomastoiditis, and 1 Central Line-associate Bloodstream Infection (CLABSI), reporting a clinical success rate of 75% [162]. Gallagher et al. treated 205 infections caused by *P. aeruginosa* MDR, primarily pneumonia (59%), and reported a clinical success rate of 74%. Interestingly, they highlighted the importance of the prompt administration of TOL-TZB (within four days), identifying it as a predictor of clinical success [163]. This observation aligns with previous studies that associated a delay in initiating effective antibacterial therapy with increased mortality in serious bacterial infections, including HAP [164,165].

An observational study involving 200 patients compared the outcomes of a TOL-TZB-based regimen versus polymyxin or aminoglycoside-based therapy [166]. In this study, a favorable clinical outcome was observed in 81% of patients in the TOL-TZB arm versus 61% of patients in the polymyxin- or aminoglycoside-based regimen arm. This difference was statistically significant.

Recently, an ASPECT-NP study involving patients with nosocomial pneumonia caused by Gram-negative pathogens was published. Pneumonia is the most frequent healthcare-associated infection acquired in the Intensive Care Unit (ICU), with high mortality rates [167,168,169]. Hospital-acquired pneumonia (HAP) can be distinguished as ventilator-associated pneumonia (VAP) or hospital-acquired ventilator-requiring pneumonia (vHAP) [170]. Within HAP, vHAP has the highest mortality [167,171].

The ASPECT-NP trial demonstrated the noninferiority of TOL-TZB to MEM for treating vHAP and VAP in both the primary endpoint of 28-day all-cause mortality and the secondary endpoint of clinical cure at the test-of-cure visit, respectively [172]. In the subgroup of vHAP, the ASPECT-NP trial also demonstrated lower mortality in the arm of patients treated with TOL-TZB compared with the arm of patients treated with MEM [172]. This result was further evaluated with multivariable analysis by Kollef et al., confirming the protective effect of TOL-TZB in this special subgroup population [173], although additional studies are needed for confirmation of these findings.

The distinct pharmacological profile of TOL-TZB, marked by enhanced activity against *P. aeruginosa*, makes it a valuable option where resistant Pseudomonas strains are prevalent or suspected [174,175].

CAZ-AVI (Zavicefta^®^) combines CAZ, a third-generation cephalosporin with potent antipseudomonal activity, and AVI, a non-BL/BLI [176]. AVI effectively neutralizes a broad spectrum of β-lactamases, including carbapenemases like KPC, OXA-48 [177], and GES [170]. Horcajada et al. reported in vitro susceptibility to CAZ-AVI ranging from 66% to 86% for MDR *P. aeruginosa* strains collected from all over the world [178].

In the literature, some studies have highlighted the efficacy of CAZ-AVI in treating various infections due to MDR *P. aeruginosa*, from cUTIs to HAP [179,180,181]. However, clinical trials involving CAZ-AVI treatments are scarce. Stone et al. reported pooled data from five Randomized Controlled Trials (RCTs), showing a favorable clinical outcome in patients treated with CAZ-AVI versus patients treated with more traditional treatment, albeit within the limitations of the study [182].

Nevertheless, the literature concerning the real-world use of CAZ-AVI is spreading. Several studies have been published, underlying positive outcomes in favor of CAZ-AVI in treating MDR *P. aeruginosa* infections [183].

The Infectious Diseases Society of America (IDSA) indicates CAZ-AVI as one of the drugs of choice in the treatment of MDR and difficult-to-treat (DTR)-*P. aeruginosa* strains, both in urinary tract infections and outside urinary tract infections, when tested susceptible [184].

FDC (Fetcroja^®^) is a siderophore cephalosporin with a unique penetration mechanism. It utilizes the iron-transport mechanism to penetrate bacterial cells, making it active against a broad spectrum of Gram-negative microorganisms, including CRP [185,186]. It is also effective against MBL. Hacket et al. demonstrated in vitro its activity against most MDR *P. aeruginosa* (99.2%), including those resistant to CAZ-AVI and TOL-TZB [187]. Lasarte-Monterrubio et al. evaluated the in vitro activity of FDC (and other novel antibiotic combinations) against strains of *P. aeruginosa* specifically resistant to CAZ-AVI and TOL-TZB [188], showing that FDC was the most active agent.

The SIDERO surveillance program, conducted between 2014 and 2019, showed a susceptibility rate to FDC of CRP strains of 99.8%, according to CLSI breakpoints [189].

The randomized APESK-cUTI demonstrated the non-inferiority of FDC versus IMP-REL in the treatment of complicated urinary tract infections in hospitalized patients for the primary endpoint of composite microbiological eradication and clinical cure at the test-of-cure visit [190].

In the APESK-NP study evaluating the efficacy and safety of FDC for the treatment of nosocomial Gram-negative pneumonia, FDC demonstrated non-inferiority to the MEM treatment. Of all the cases, *P. aeruginosa* represented the second most common pathogen in the FDC arm. Clinical success was achieved in 67% of pneumonia cases caused by *P. aeruginosa*, without statistical difference from the pneumonia cases caused by *P. aeruginosa* in the MEM arm [191].

However, further studies on its efficacy in real life are needed to better assess its effectiveness.

IMP-REL (Recarbrio^®^) combines IMP, a carbapenem approved in 1985, and REL, a BLI that enhances IMP’s activity by protecting it from enzymatic degradation. This combination is active against class A and class C β-lactamases. On the other hand, it is ineffective against OXA-48 and MBLs [192].

The SMART study, a surveillance study conducted in several countries across the world, assessed the susceptibility of IMP-REL [193]. Furthermore, Lob et al. showed that the addition of REL restored the activity of IMP against *P. aeruginosa* strains resistant to IMP alone.

Mushtaq et al. collected *P. aeruginosa* clinical strains producing ESBL and Carbapenemases, showing an 80.5% susceptibility for IMP-REL [194]. In this study, the main mechanism of resistance was the production of beta-lactamases not susceptible to inhibition by REL, such as MBLs, OXAs, and GES.

In the RESTORE-IMI clinical trial, IMP-REL demonstrated greater efficacy than COL/MEM against *P. aeruginosa* infection (81% vs. 63%). However, this difference was not statistically significant due to the small sample size [195].

The RESTORE-IMI 2 clinical trial, a randomized, double-blind controlled trial, was conducted to assess the efficacy of IMP-REL in adult patients with HAP/VAP [196]. IMP-REL was non-inferior to the comparator (PIP-TZB) for both endpoints (day 28 all-cause mortality and favorable clinical response at early follow-up). In this study, *P. aeruginosa* was the second most abundant pathogen isolated.

IDSA guidelines recommend IMP-REL as one of the drugs of choice in the treatment of cUTIs and infections outside the urinary tract due to DTR *P. aeruginosa* strains when tested as susceptible [184].

In addition, promising antibiotics are coming into the pipeline, such as cefepime/enmetazobactam, cefepime/zidebactam, cefepime/taniborbactam, and plazomicine.

These FEP-based antibiotics had broad-spectrum activity against Gram-positive and Gram-negative bacteria. The distinguishing factor is the specific BLI paired with FEP to shield it from enzymatic degradation. While all four inhibitors fall under the category of next-generation BLI, Enmetazobactam is classified as a penicillanic acid sulfone, Taniborbactam as a boronic acid derivative, and Zidebactam as a diazabicyclooctane [197]. Their effectiveness is still being evaluated, but the clinical trials have yielded promising results [198,199,200]. However, no real-life studies compare these three molecules [201]. J Vázquez-Ucha et al. assessed the in vitro efficacy of these molecules, finding that cefepime/zidebactam was the most potent combination against carbapenemase-producing Enterobacterales, followed by cefepime/taniborbactam and cefepime/enmetazobactam. Furthermore, Moya et al. highlighted that zidebactam alone has significant activity against *P. aeruginosa*. This effect is due to the inhibition of PBP2, leading to the creation of spheroplasts, the disruption of the outer membrane, and, as a result, protection against common membrane-bound resistance mechanisms exhibited by *P. aeruginosa* [197].

Plazomicin is a next-generation aminoglycoside antibiotic synthetically derived from sisomicin [202]. It works by inhibiting bacterial protein synthesis, leading to dose-dependent bactericidal activity. One of its significant advantages is its activity against bacterial strains harboring clinically relevant AMEs [203]. However, it is worth noting that plazomicin is not active against bacterial isolates expressing ribosomal methyltransferases, which can lead to aminoglycoside resistance [203]. Focusing on *P. aeruginosa*, plazomicin has shown promising data [204]. The antibiotic demonstrates synergistic activity when combined with other agents like FEP, Doripenem, IMP, or PIP/TZB [205].

## 5. Conclusions

Managing *P. aeruginosa* infections remains a formidable challenge in the clinical setting, primarily due to the bacterium’s intrinsic and acquired resistance mechanisms. While traditional monotherapies have served as the cornerstone of treatment, the rise of multidrug-resistant strains has necessitated a shift towards combination therapies and the development of new antibiotics. The judicious use of antibiotics, guided by accurate antibiograms, is paramount. Understanding the myriad of resistance mechanisms employed by *P. aeruginosa* is essential for the effective treatment of patients. The correct interpretation of antibiograms allows for the selection of the most suitable antibiotic therapy, enhancing patient outcomes and minimizing the risk of treatment failure. Conversely, misinterpretation can lead to inappropriate antibiotic use, inadvertently promoting the selection of resistant strains and exacerbating the global issue of antibiotic resistance. Such mistakes jeopardize individual patient outcomes and have broader implications for public health. As the battle against *P. aeruginosa* continues, this review aims to equip readers with the knowledge to interpret antibiograms accurately, fostering better clinical decision-making. Further studies are essential to elucidate the efficacy of new antibiotics against *P. aeruginosa*, enabling a deeper understanding of how to strategically deploy these drugs against this resilient bacterium. Such knowledge is pivotal to prevent the emergence of new resistance mechanisms, ensuring the sustained effectiveness of our therapeutic arsenal.

## Figures and Tables

**Table 5:** Enzyme types and their activity against antibiotics.

Class	Active Site	Enzyme Type	Substrates
A	Serine	Penicillinases:	
		Broad-spectrum	Benzylpenicillin, aminopenicillins, carboxypenicillins, ureidopenicillins, narrow-spectrum Cephalosporins
		Extended-spectrum (β-lactamase)	Substrates of broad-spectrum plus oxymino-β-lactams (cefotaxime, ceftazidime, ceftriaxone) and aztreonam
		Carbapenemases	Substrates of extended-spectrum plus cephamycins and carbapenems
B	Zn^2+^	Carbapenemases	Substrates of extended-spectrum plus cephamycins and carbapenems
C	Serine	Cephalosporinases	Substrates of extended-spectrum plus cephamycins
D	Serine	Oxacillinases:	
		Broad-spectrum	Aminopenicillins, ureidopenicillin, cloxacillin, methicillin, oxacillin, and some narrow-spectrum cephalosporins
		Extended-spectrum (β-lactamase)	Substrates of broad-spectrum plus oxymino-β-lactams and monobactams
		Carbapenemases	Substrates of extended-spectrum plus cephamycins and carbapenems

**Table 6:** ESBL variants in *P. aeruginosa* and their MICs against principal antibiotics.

Strain	ESBL	MIC (mg/L)									
		IMP	MEM	CAZ	CAZ-AVI ^a^	CAZ-AVI ^b^	CAZ-AVI ^c^	TOL-TZB ^d^	TOL-TZB ^e^	TOL-TZB ^f^	PIP-TZB
R1189	GES-1	1	2	16	2	2	2	32	32	16	16
R184	GES-2	16	32	64	4	2	2	16	8	4	128
R186	GES-5	32	128	64	16	8	8	8	8	8	128
R3451	GES-6	16	64	32	2	2	2	32	32	32	64
R1188	CTX-M	1	8	32	8	4	4	4	1	1	32
R1192	PER-1	0.5	1	>512	64	16	16	512	128	64	64
R1185	BEL-1	1	2	32	4	2	2	8	8	8	64
R1187	BEL-2	0.5	2	128	8	4	2	32	32	32	128
R1205	VEB-1	8	16	256	8	4	4	64	32	32	32
R1217	TEM-4	1	0.5	8	2	1	1	0.5	0.5	0.5	4
R136	SHV-2a	1	2	32	4	4	2	4	4	2	256

IPM, imipenem; MEM, meropenem; CAZ, ceftazidime; CAZ-AVI, ceftazidime/avibactam; TOL-TZB, ceftolozane/tazobactam; PIP-TZP, piperacillin/tazobactam.^a^ Avibactam at 4 mg/L, ^b^ Avibactam at 8 mg/L, ^c^ Avibactam at 16 mg/L.^d^ Tazobactam at 4 mg/L, ^e^ Tazobactam at 8 mg/L, ^f^ Tazobactam at 16 mg/L.

**Table 7:** Phenotypic antibiogram of clinical strain *P. aeruginosa* compatible with a GES enzyme expression.

*P. aeruginosa*	Rectal Swab	
Antibiotic	MIC	SIR *
Amikacin	≤4.0	S
Cefepime	>16.0	R
Ceftazidime	>64.0	R
Ceftazidime/Avibactam	=8.0	S
Ceftolozane/Tazobactam	=64.0	R
Ciprofloxacin	=0.5	I
Colistin	=1.0	S
Meropenem	=16.0	R
Piperacillin/Tazobactam	>128.0	R

MIC = minimum inhibitory concentration; SIR = Susceptible (S), Susceptible, Iincreased Eexposure (I), and Resistant (R).* Breakpoint sec. Clinical breakpoints (v 13.1) [44].Comment: pPhenotype resistant to cefepime, ceftazidime, piperacillin/tazobactam, meropenem and ceftolozane/tazobactam. The phenotype could be compatible with a GES enzyme expression.

**Table 8:** The use of genetic technique sequencing plus the phenotypic antibiogram. The GeneXpert^®^ via PCR method detected VIM. The phenotypic antibiogram correlates with the MBL detected.

*P. aeruginosa*	Blood Culture	
**GeneXprt^®^**		
KPC	Not detected	
VIM	**Detected**	
IMP	Not detected	
NDM	Not detected	
OXA-48	Not detected	
**Antibiotic**	**MIC**	SIR *
Amikacin	=16.0	S
Cefepime	>16.0	R
Ceftazidime	>64.0	R
Ceftazidime/Avibactam	>64.0	R
Ceftolozane/Tazobactam	>64.0	R
Ciprofloxacin	>1.0	R
Colistin	=1.0	S
Meropenem	>64.0	R
Piperacillin/Tazobactam	>128.0	R
Cefiderocol	≤2.0	S

MIC = minimum inhibitory concentration; SIR = Susceptible (S), Susceptible, Iincreased Eexposure (I), and Resistant (R). * Breakpoint sec. Clinical breakpoints (v 13.1) [44]. Comment: tThe antibiogram shows a resistance profile to all BL and new BL/BLI. Cefiderocol is susceptible. PCR detected VIM. This phenotype is compatible with the VIM MBL *P. aeruginosa* strain.

**Table 9:** Phenotypic antibiogram of clinical strain *P. aeruginosa* compatible with the production of AmpC enzyme.

*P. aeruginosa*	Ecoculture	
Antibiotic	MIC	SIR *
Amikacin	=8.0	S
Cefepime	=4.0	I
Ceftazidime	=16.0	R
Ceftazidime/Avibactam	=2.0	S
Ceftolozane/Tazobactam	=1.0	S
Ciprofloxacin	=0.125	I
Colistin	=1.0	S
Meropenem	=0.25	S
Piperacillin/Tazobactam	=32.0	R

MIC = minimum inhibitory concentration; SIR = Susceptible (S), Susceptible, Iincreased Eexposure (I), and Resistant (R).* Breakpoint sec. Clinical breakpoints (v 13.1) [44].Comment: tThe antibiogram shows a resistance profile to ceftazidime and piperacillin/tazobactam. Instead, cCefepime is still intermediate. Meropenem and new BL/BLIs are susceptible. This phenotype is compatible with the AmpC *P. aeruginosa* strain.

**Table 10:** Phenotypic antibiogram of clinical strain *P. aeruginosa* shows mutated AmpC enzyme production.

*P. aeruginosa*	Bronchoalveolar Lavage	
Antibiotic	MIC	SIR *
Amikacin	≤4.0	S
Cefepime	=8.0	I
Ceftazidime	=64.0	R
Ceftazidime/Avibactam	=64.0	R
Ceftolozane/Tazobactam	=64.0	R
Ciprofloxacin	=0.5	I
Colistin	≤0.5	S
Meropenem	=8.0	I
Piperacillin/Tazobactam	=16.0	R

MIC = minimum inhibitory concentration; SIR = Susceptible (S), Susceptible, Iincreased Eexposure (I), and Resistant (R). * Breakpoint sec. Clinical breakpoints (v 13.1) [44]. Comment: pPhenotype resistant to ceftazidime, piperacillin/tazobactam, and new BL/BLIs. Increasing MIC of cefepime and meropenem. The phenotype could be compatible with an AmpC mutation in *P. aeruginosa* strain.

## Data Availability

No new data were created or analyzed in this study. Data sharing is not applicable to this article.
